# The Development and Evaluation of Netball-Specific High-Intensity Interval Training Sessions: The Netball-HIIT Study

**DOI:** 10.3390/sports12010034

**Published:** 2024-01-18

**Authors:** Narelle Eather, Katelyn Stansfield, Mark Babic, David R. Lubans

**Affiliations:** 1 Centre for Active Living and Learning, University of Newcastle, Callaghan, NSW 2308, Australia; katelyn.stansfield1@det.nsw.edu.au (K.S.); mark.babic@newcastle.edu.au (M.B.); david.lubans@newcastle.edu.au (D.R.L.); 2 College of Human and Social Futures, University of Newcastle, Callaghan, NSW 2308, Australia; 3 Hunter Medical Research Institute, New Lambton Heights, NSW 2305, Australia; 4 Faculty of Sport and Health Sciences, University of Jyväskylä, 40014 Jyvaskyla, Finland

**Keywords:** coaching, fitness, HIIT, high intensity, netball, sport, injury prevention

## Abstract

This two-phase study involved the design, development, and evaluation of netball-specific high-intensity interval training sessions (Netball-HIIT) for use with netballers of varied ages and abilities. In Phase 1 (2020), a systematic analysis of netball GPS data from 30 netball players and gameplay video footage (10 h) was conducted, followed by the design and testing of five 8 min Netball-HIIT sessions involving 100 netball players (age 21 ± 8.44 years; Australia). In Phase 2 (2021), the feasibility and preliminary efficacy of delivering one Netball-HIIT session each week for five weeks was assessed using a two-armed, dose-matched, randomized, controlled feasibility study with netball players (born in 2010) (Netball HIIT n = 15; Netball Knee Program: NKP n = 15). Cardiorespiratory and muscular fitness was assessed at baseline and 6 weeks. Data were analyzed using linear mixed models and Cohen’s *d* effect sizes. Netball-HIIT sessions were highly rated by players, and higher average (139 bpm) and peak heart rates (156 bpm) were detected amongst Netball-HIIT participants (KNP = 127 bpm and 152 bpm). We observed a large effect for cardiorespiratory fitness (+2.4 laps, *d* = 0.89), and a small to medium effect for muscular fitness (push-ups +1.2, *d* = 0.49; standing jump +0.8 cm, *d* = 0.36) in favor of Netball-HIIT, suggesting that coach-led Netball-HIIT may provide a time-efficient and effective training component for use in netball.

## 1. Introduction

Participation in individual and team sports has shown to be beneficial to physical, social, psychological, and cognitive health outcomes [1,2,3], and these benefits often provide additional or more potent health benefits than those achieved through other types of physical activities. The public health benefits of participating in sports are far-reaching, given that ~70% of children and adolescents and up to 40% of adults participate in some form of individual or team sport [4,5].

Sports provide athletes with an opportunity to engage in high-intensity or vigorous physical activity (VPA), which activates several complex physiological responses and contributes to the development and maintenance of cardiorespiratory fitness (CRF) [6]. CRF is important for participating in and succeeding in many individual and team sports and provides individuals with a multitude of health benefits [7]. Furthermore, the development of muscular fitness (strength, endurance, and power) is also important for motor skill proficiency in many sports and is linked to a range of short and long-term health outcomes and reduced injury risk [8,9,10]. However, in Australia, some community/club level sport coaches do not have formal qualifications, are often parent volunteers [11,12], and sometimes lack the knowledge and expertise to maximize opportunities for all participants to engage in health-enhancing doses of VPA. In addition, coaches do not always have strategies for planning and delivering sports sessions that are enjoyable, engaging, and motivating for participants [13]. In doing so, training sessions may fail to develop sport-specific fitness and, therefore, limit children’s success and/or enjoyment in sports participation. For example, previous studies show that children can spend up to two-thirds of sports training session time sedentary or engaged in light physical activity (PA) or as little as 20 min out of 60 in moderate-to-vigorous physical activity (MVPA) [14]. Providing coaches with session ideas that maximize players’ PA during training, may help to simultaneously achieve fitness and motor skill objectives.

High-intensity interval training (HIIT) is a popular and time-efficient form of exercise [15]. HIIT consists of short but intense (from ≤45 s to 2–4 min), repeated bouts of activity (typically >85% of age-predicated maximal heart rate), separated by short periods of active rest or recovery [16,17]. HIIT has demonstrated the capacity for enhancing a range of health outcomes (including physical fitness, cognition, cardiovascular disease biomarkers, and mental health) in varied populations, including children, adolescents, and adults, in various settings (e.g., education, workplace, sport, exercise settings) [18,19,20].

Embedding HIIT into sport training sessions represents a novel approach for increasing the dose of VPA that sports participants receive during training [21]. This strategy may have utility if sessions are (1) specifically designed to target and develop the fitness and motor skill demands of the chosen sport; (2) they are relatively simple and can be implemented by volunteer coaches (requiring minimal preparation, equipment, or time); and (3) they can be easily adapted to suit the needs of different players and teams (to maximize enjoyment, motivation, and engagement). Lending itself to HIIT training is the popular team sport, netball. Netball is played by two opposing teams of seven players on a rectangular court with the objective of scoring goals by shooting it through a netball ring (3.05 m high) from within the defined area known as the netball circle. Players are assigned specific positions and are restricted to move within certain areas of the court, with games lasting 60 min in duration. During a netball game, players are required to perform short bursts of high-intensity activity (running, sprinting, dodging, and jumping), accompanied by periods of reduced effort (clearing movements, passing, and holding). However, significant variations in heart rate are typical across playing positions, with available literature suggesting that the Goal Shooter (GS) and Goal Keeper (GK) are the only two positions to spend significant amounts of time in the moderate intensity zone (70%HRmax); whereas the Centre (C), Goal Attack (GA), and Goal Defence (GD) have the greatest cardiovascular demands, spending substantial amounts of time engaged in vigorous-intensity activity (>85%HRmax), predominantly due to the greater court coverage required by these positions [22]. Additionally, the quantity of time spent in the MVPA or VPA (>70%HRmax) ranges from 7% to 62.6% across positions throughout the game, emphasizing a distinct difference in CRF requirements of netball players during match play [22]. Despite this knowledge, there is limited research specifically exploring the effectiveness of embedding HIIT into sports training sessions, especially sport-specific HIIT sessions [23]. Therefore, the main objective of our two-phase study was to (1) design and develop netball-specific HIIT (Netball-HIIT) sessions and (2) assess the feasibility and preliminary efficacy of Netball-HIIT for improving cardiorespiratory and muscular fitness when compared to a recommended preparation and conditioning program for netball.

## 2. Materials and Methods

**Figure 1:**
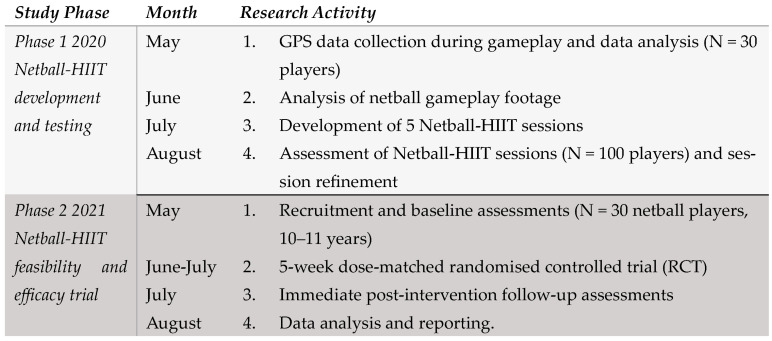
Netball-HIIT study timeline.

We conducted the Netball-HIIT study in two distinct phases. Phase 1 (March–August 2020) involved the design of five Netball-HIIT sessions, and Phase 2 (May–August 2021) involved the evaluation of the 5-session Netball-HIIT program using a dose-matched two-armed randomized controlled trial involving junior netball players (see Figure 1). Ethics approval for the entire study was provided by the University of Newcastle Human Research Ethics Committee. Study participants provided written informed consent prior to participation in each phase of the study. Trial Registration: Australian New Zealand Clinical Trials Registry: ACTRN12620001219976.

### 2.1. Phase 1: Netball-HIIT Session Design and Evaluation 

#### Recruitment, Participants, and Processes

*Step 1: gameplay GPS data collection:* Twenty experienced female netball players (aged between 14 and 42 years) were recruited to participate in the design phase of the study. Upon providing written informed consent, eligible netball players were required to participate in two × 60 min games of netball conducted one week apart at the community netball courts (The Hunter Region, NSW, Australia) wearing a GPS tracking device (SPT2-GPS tracker) (Game Traka, 2020). Eligible participants had to be registered with a club or representative netball team and without existing medical conditions or injuries preventing participation in match play. All GPS devices were set up and positioned in the pocket of the GPS vest by a member of the research team and collected the following variables: maximum and average running speed (m/s), total distance covered (m), and movement patterns on court. At the completion of the game, all GPS data were downloaded using the SPT bridge to GameTraka for analysis.

*Step 2: Gameplay video analysis:* Video analysis was used to investigate specific movement patterns frequently used by netball players (including movement types, angles, patterns, and distances) across positions and age groups. Ten hours of video footage of netball games involving adolescent representative players (provided by The Regional Academy of Sport, Australia), adult club level players (broadcast by NBN), and adult representative premier league players (live-streamed by BarTV) from the 2019 to 2020 seasons were observed and coded play-by-play by one member of the research team (with 10% coded independently by a second member and checked for accuracy). The predominant movement patterns were categorized as sprinting, jogging (sub-maximal running), clearing (moving out of the immediate play area), dodging, jumping, or side-stepping. 

*Step 3: Netball-HIIT session design:* Guided by the GPS data and video analysis and the protocols used in our previous HIIT studies with adolescents and adults [24], the research team designed a 2 min general warm-up and five Netball-HIIT sessions lasting eight minutes (30 s:30 s work/rest ratio). Each session included a combination of aerobic exercises and muscle-strengthening exercises that incorporated netball-specific movements, fitness requirements, and motor skills (final HIIT sessions can be accessed by contacting the corresponding author). 

*Step 4: Pilot testing of Netball-HIIT sessions:* One hundred female adolescent and adult netball players from (12 teams; mean age 21.07 ± 8.44) were recruited to evaluate the Netball-HIIT sessions (July 2020). Eligible participants provided written informed consent prior to participating in the designed two-minute warm-up and one of the eight-minute Netball-HIIT sessions (30 s:30 s work/rest ratio) whilst wearing a Polar H10 heart rate monitor (Heart Rate Sensors Collection|Polar Australia) to track mean and peak heart rate during HIIT sessions. Eligible participants were females registered with a club or representative netball team and had no existing medical conditions or injuries preventing participation in the Netball-HIIT session. Players completed the Netball-HIIT sessions in small groups/or as a team, with a member/s of the research team directly observing each session. To ensure end-users were involved in the development of our tailored HIIT sessions, verbal and written feedback was sought from participants to determine session suitability for netball, comprehension of the session task cards, and enjoyment of the session. Heart rate monitoring using Polar HR monitors synced to the Polar Team tablet application (app) was also used to investigate intensity of the workouts and determine if the sessions replicated the intensity experienced during a netball game. 

### 2.2. Phase 2: Netball-HIIT Implementation and Evaluation 

#### 2.2.1. Recruitment, Participants, and Processes

We conducted a two-armed, dose-matched, randomized, controlled feasibility study in 2021 involving 30 female junior representative-level netball players born in 2010 (turning 11 in 2021) with no existing medical conditions or injuries preventing participation in HIIT. Assessments were conducted at baseline (May 2021) and immediately post-intervention (June 2021) at the club’s outdoor netball courts by the research team. Participants were recruited as individuals and then, after baseline assessments, allocated to one of two study conditions (Netball-HIIT or NKP [25]) by an independent person from the Netball Association using a Microsoft Excel list of participant study numbers. 

#### 2.2.2. Treatment Conditions

(1) Netball-HIIT program: Players randomized to Netball-HIIT conditions completed a 5-week intervention delivered in the first 10 min of training by one Representative coach and supervised by a member of the research team. As a group (and split into pairs by choice), participants were asked to complete a set 2 min warm-up and one Netball-HIIT session (after which they completed the remainder of their 2 h training session). Each of the five Netball-HIIT sessions required players to work in pairs for 8 min, with a work-to-rest ratio of 30 s:30 s.
(2) Netball Knee Program: Players randomized to the active control group received Netball Knee Program (NKP) [25], which is a free netball-specific injury prevention and conditioning program accessible and promoted to all netball coaches in Australia. The program is designed for use at the start of netball training sessions and specifically targets the preparation and conditioning of players (junior to elite) to perform rapid acceleration and deceleration, sharp changes in direction, jumping, and landing. In this study, 10 min of the NKP was implemented at the start of training over five weeks by one Representative coach and supervised by a member of the research team.

Participants in both groups wore Garmin GPS and Heart Rate monitoring wrist-worn devices to record heart rate (beats per minute; average and maximum) and distance (kilometers) achieved in each 10 min session. Players were verbally encouraged to work to capacity during each activity (with a target heart rate of >85%_max_ set for Netball-HIIT) to ensure appropriate exercise intensity was reached and maintained, and skills were developed under game-like conditions across the five sessions. 

#### 2.2.3. Measures

The following outcomes were assessed by a member of the research team:

1. Process evaluation: Assessment of
  (i) Compliance and attendance (i.e., participants’ attendance and completion of scheduled sessions in real-world environment) assessed using an attendance roll;
  (ii) Fidelity (adherence to session protocols) measured using heart rate data (collected by a wrist-worn heart rate and GPS Garmin device worn in the first 10 min of training and completion of programmed Netball-HIIT sessions;
  (iii) Satisfaction (via questionnaire assessing participants’ enjoyment of HIIT adapted from Paxton’s (2008) physical activity enjoyment scale: Netball HIIT group only) completed at follow-up.
2. *The Affective/Feelings State questionnaire* [26]: a one-item questionnaire administered pre- and post-HIIT sessions asking participants to respond to the question ‘How are you feeling right now?’ (−5 = very bad to +5 = very good) following each HIIT session.
3. *Efficacy outcomes: Physiological measures*: At baseline and follow-up using standardized published protocols (i) CRF (20 m Shuttle Run Test) [27] with total number of 20 m laps recorded and (ii) muscular fitness (90^0^ Push-Up test [27], standing long jump). A single attempt of the 90^0^ Push-Up test was completed (with total complete push-ups recorded), and participants completed the standing broad jump twice (with the highest distance score recorded in meters).

*Psychological measures:* (i) The High-Intensity Interval Training Self-efficacy Questionnaire (HIIT-SQ) (Netball-HIIT group only) [28] using 6 items and a 10-point Likert scale (0 = Not at all confident; 10 = Completely confident) was administered at follow-up.

## 3. Statistical Analyses

Statistical analyses were conducted using IBM SPSS Statistics for Windows (Version 20) (SPSS, INC 2010, IBM Company, New York, NY, USA). We did not perform a power calculation as this was a program development and feasibility study, although a recruitment target of 100 (Phase 1) and 30 (Phase 2) was set. Phase 1 and Phase 2 feasibility data were investigated using descriptive statistics (e.g., means, percentages). Linear mixed models (for fitness outcomes) and one-way ANOVA (for heart rate and GPS data) were used to assess differences in mean scores between treatment conditions (Netball-HIIT or Knee Program), and Cohen’s *d* was calculated by dividing the mean difference in change by the pooled standard deviation of change for each variable [29]. 

## 4. Results

### 4.1. Phase 1 Results

*Results Step 1: gameplay GPS data collection*: The GPS data indicated that the average top speed for the netball participants during gameplay ranged from 9.50 km/h to 26.25 km/h, and the average distance covered ranged from 2.04 km to 11.67 km across the two × 60 min periods. 

*Results Step 2: gameplay video analysis:* Most movements performed during the analyzed video footage of netball gameplay were less than ten seconds in duration, followed by approximately 30 s of recovery. All movement patterns and the duration of work efforts were similar for all playing levels. A summary of the movement analysis is provided in Table 1. 

*Results Step 3: Netball-HIIT session design:* Informed by the data collected from GPS tracking and Video analysis, five HIIT sessions specific to netball were designed. The protocols for the Netball-HIIT sessions aligned with the research team’s previous HIIT studies (including Uni HIIT, Work HIIT, Burn2Learn, and HIIT for Teens) and included a combination of aerobic and muscular fitness activities performed in a 30 s:30 s rest-to-work ratio. The Netball-HIIT task cards can be accessed by emailing the corresponding author. In summary:

Netball-HIIT 1 focused predominantly on attacking movements (e.g., driving forward and at angles) and included jumping and core strength activities.
Netball-HIIT 2 had a predominantly defensive movement focus, including footwork activities and whole-body and core strength activities (such as push-ups and lunges).
Netball-HIIT 3 had an attacking focus using dynamic, explosive drives and movements to target agility and power, along with the inclusion of core and leg strength activities.
Netball-HIIT 4 had a core and upper body strength and explosive movement focus, including lower body power exercises (e.g., high skips).
Netball-HIIT 5 provided activities targeting attack and defending fitness requirements using a combination of power-, agility-, and speed-related movements, including explosive angled and straight drives, dodges, and lateral jumps.

*Results Step 4: Pilot testing of Netball-HIIT:* A total of 100 netball players (age range of 12–53 years: mean age of 21 ± 8.44 years) participated in heart rate tracking while completing the five Netball HIIT sessions. For the percentage of heart rate maximum achieved across sessions, there was a significant difference observed by age group, playing level, and HIIT session (Table 2). 

The qualitative verbal feedback provided by players (N = 21; 10 representative levels, 10 club level) after Netball-HIIT sessions indicated that most players enjoyed the sessions, felt that they were beneficial for netball conditioning, and the activities were well suited to and reflective of the movement requirements of netball. Player statements included: “*I really enjoyed the HIIT sessions. They were quick to do and involved lots of different exercises which I liked as it kept it interesting, especially some involving two elements (e.g., squat jumps and dodges)*” (Representative player A); “*As I am a mid-court player, I feel the exercises suit where I play really well, as they involved lots of footwork activities and burst of high-intensity efforts*” (Representative player B); “*I really enjoyed all of the short interval sessions, they were suitable to all positions. Set out at a good pace to get the heart rate up with relevant exercises for game play and they incorporated injury prevention exercises too*” (Club player A). However, one participant reflected negatively on the various sit-up exercises due to a hard surface causing discomfort, particularly the club athletes training on an outdoor court. This was also observed by the research team. Example player feedback included: “…*The only activity I didn’t enjoy was the sit-ups as I didn’t have a towel to make the surface a bit softer, all other activities were great and helpful for my court fitness*” (Club Player B). Based on the feedback, minor modifications to the Netball-HIIT sessions were made (and implemented in Phase 2) to maximize the player enjoyment of each session, the performance, and the suitability of sessions for netball performed on both indoor and outdoor surfaces. 

### 4.2. Phase 2 Results 

*Compliance* All five sessions were conducted; however, there was a break between weeks four and five due to weather and training being canceled. 

*Attendance*: On average, 27/30 participants attended all sessions across the five weeks (90%). Three participants were absent for one training session dues to sickness. Injuries sustained during scheduled games hampered full participation for some participants (e.g., shin splints, rolled ankle, broken arm), and modifications were provided accordingly. No injuries occurred during the delivery of either program. 

*Fidelity:* During each session, both heart rate and affective feeling states were monitored and recorded (with valid heart rate data recorded) (See Table 3). Averaged across all HIIT sessions, participants in the Netball-HIIT had an average heart rate of 139 bpm or 67%HR_max_ (including rest and work intervals) and peak heart rate of 156 bpm or 75%HR_max_, and KNP had an average of 127 bpm or 61%HR_max_ and a peak of 152 bpm or 73%HR_max_. Significant differences between groups (*p* < 0.05) were observed for average heart rate in Session 1, 4, and 5 as well as for peak heart rate in Session 1. 

Additionally, across the 8-week program, the feeling state survey indicated that there was no significant change in affective state across sessions for either group. Players in the Netball-HIIT group had a decline of −0.2, and players in the NKP had an increase of +0.02 regarding the pre-post rating (Table 1). *Enjoyment*: Participants in the Netball-HIIT program reported an average enjoyment of HIIT score of 11.60 ± 5.49 (with possible scores ranging from 7 to 35 and a low score indicating positive enjoyment). 

#### Preliminary Efficacy Results

*Physiological outcomes:* We observed a large positive effect for CRF (+2.4 laps, 95% CI (1.54–20.91); *d* = 0.89), and small-to-medium effects resulted for muscular fitness (push-ups +1.2, *d* = 0.49; standing jump +0.8 cm, *d* = 0.36) (Table 4). *Psychological outcomes*: Netball-HIIT participants reported that their HIIT Self-efficacy was 45.36 ± 7.76 (out of 60) when rating themselves at the completion of the 5 weeks.

## 5. Discussion

The aim of our study was to design, develop, and evaluate netball-specific high-intensity interval training sessions. We found that the Netball-HIIT sessions successfully targeted netball movement patterns, motor skills, and physical fitness requirements. The sessions invoked heart rate intensity levels of ≥75%_max_HR and facilitated improved cardiorespiratory and muscular fitness in players when integrated into the start of training sessions once per week. Our findings provide initial support for Netball-HIIT as a time-efficient and effective training component suitable for use in netball.

Improving the health status of our population is a global priority, with PA being a core element of attaining and maintaining optimal health. Sports, a subset of physical activity, has shown to be particularly beneficial for facilitating a range of positive physical, mental, and social health outcomes [3,30]. Therefore, sports coaches are perfectly positioned to help participants develop movement patterns and motor skill competencies, which are important for lifelong PA participation. Importantly, when sports coaches engage their players or athletes in MVPA/VPA and muscle-strengthening exercises, the greatest health benefits can be achieved [7,8,9,10]. However, research demonstrates that sport training sessions are largely inactive [14,31], and many coaches are untrained and/or inexperienced volunteers who often seek help in planning and delivering effective sports training sessions [11]. By providing netball coaches with a novel and evidence-based PA program, we are targeting a public health issue and maximizing training effectiveness and quality (by targeting netball-specific movement skills and fitness) largely without impacting limited training time [13].

The Netball-HIIT study aligns directly with three key considerations presented in the conceptual model recently published by Lubans and colleagues (2022) [21]. Lubans et al. advocate that there are four complementary elements vital for successfully designing and delivering HIIT sessions for adolescents that target population health at scale. The conceptual model highlights the importance of (1) incorporating HIIT into existing PA opportunities (e.g., sports sessions); (2) developing physical literacy through HIIT sessions (e.g., increased confidence and competence to perform sport-related movement patterns and skills through participation in Netball-HIIT); and (3) delivering HIIT in an engaging manner (e.g., using a variety of exercises directly targeting improved performance in a sport of choice). These tenets have been successfully targeted in previous HIIT intervention studies [24,32]; however, this is the first study to report on the design and evaluation of a HIIT program targeting these elements in organized sports (i.e., netball). The next step in the research continuum, for long-term impact at scale, is the fourth tenant, which will need to be adopted in a future large-scale study, whereby systematic guidance and support is provided to netball coaches delivering Netball-HIIT to teams within clubs who may face varied barriers and challenges (e.g., coaching confidence/experience, player motivation).

The success of Netball-HIIT in facilitating greater improvements in cardiorespiratory and muscular fitness when compared to a recommended netball preparation and conditioning program supports the growing body of evidence supporting the effectiveness of HIIT for children and adolescents [23]. Importantly, improvements in fitness (after only five low-dose Netball HIIT sessions conducted once per week) demonstrate that sports coaches can have a public health impact with only small changes to their training sessions. In this study, coaches simply re-allocated the 10 min warm-up (typically devoted to gross motor activities, conditioning, and dynamic stretching) to Netball-HIIT. Furthermore, the Netball-HIIT sessions were provided on easy-to-follow task cards to reduce coach preparation and organization. Players simply followed the task cards, and coaches provided encouragement, technical advice, and positive feedback throughout sessions. This approach has also been successful in the school setting, with teachers re-allocating time at the start of their physical education, sports, or academic lessons for the inclusion of HIIT, with findings supporting overall small–medium improvements to cardiorespiratory and muscular fitness [23]. Similar to this study, HIIT has also been shown to be more potent in facilitating improvements in fitness outcomes in the school setting when compared to control or active comparison groups (such as games and sports, stretching, moderate-intensity activity, and normal practice) [23]. However, previous studies have not compared two sport-specific programs; instead, comparisons have been made between HIIT programs and treatment conditions involving cycling, running, body weight/resistance training/plyometric exercises, games and sports, or combinations of these activities.

HIIT protocols generally include aerobic exercises performed at a target intensity of 85–100% of the age-predicated maximal heart rate [21]. Recently, adaptations of HIIT have been used in “real-world” settings (such as schools, workplaces, and sports), which include a variety of exercises (such as muscular fitness exercises, ball activities, and dance) to provide a more palatable and enjoyable PA experience [20,21,23]. The average peak heart rate (75%maxHR) experienced by players in this study is not considered VPA and may have resulted from (1) the variety of exercises used in the Netball-HIIT sessions (muscular fitness and aerobic), with the muscular fitness exercise being less intense (e.g., lunch walks), or (2) the inexperience of the players with performing HIIT. With increased exposure to HIIT and mastery of the movement patterns and core exercises included in Netball-HIIT, players’ ability to push themselves or perform more challenging extensions of included activities (e.g., squat jumps vs. squats, full push-ups vs. modified pushups) is likely to occur and result in higher levels of intensity.

This two-phase study saw the design, evaluation, and implementation of Netball-HIIT. Our results provide preliminary evidence to support the acceptability and efficacy of the five-session high-intensity interval training program “Netball-HIIT” when implemented by netball coaches at the start of netball training with a sample of 11-year-old netball players and add to the growing body of evidence supporting the feasibility and acceptability of tailoring HIIT protocols for participants and implementing them in ‘real-world’ settings (beyond the laboratory), such as workplace [20], educational, and sports settings [23], for healthy and unhealthy populations [32]. The major strengths of this study include the novelty of the Netball-HIIT program, its data-informed design, the inclusion of club and elite-level participants in the program development, and the use of a randomized controlled trial design. Limitations of the study included the small sample, single location, and single age group involved in the Netball-HIIT trial. Further, participant’s maturity status, body composition, and habitual physical activity behaviors were not measured, limiting our understanding of whether these factors influenced study findings. Building the evidence to support the acceptability and effectiveness of the Netball-HIIT and other sport HIIT variations across various age groups, skill levels, and sports settings is required.

## 6. Conclusions

Providing sports coaches with a range of user-friendly and evidence-based strategies and resources for increasing the amount and intensity of PA in sports training sessions and developing the fitness and skill capacities of their players and athletes may be an important step in developing the quality of sports training sessions. This may be especially useful for sports coaches at the community/club level of organized sports who may be inexperienced or lack access to professional development and resources regarding the planning and delivery of high-quality sports sessions.

## Figures and Tables

**Table 1:** Step 1: netball gameplay GPS data (Australia, 2021).

Players Position	Average Top Speed	Average Distance Covered (120 min)	Average Distance in Hard Running
Midcourt (WA, C, WD)	23.70 km/h	8.26 km	16.3 m
Shooters (GS, GA)	22.70 km/h	6.90 km	10.8 m
Circle Defenders (GD, GK)	17.71 km/h	5.29 km	6.8 m

**Table 2:** Percentage of heart rate maximum (average) by subgroup.

Subgroup	Percentage of Heart Rate Maximum (Average)
Age	
*Adolescents*	83 ± 5.60%HR_max_
*Adults*	80 ± 7.12%HR_max_
Playing Level	
*Representative*	81 ± 6.48%HR_max_
*Club*	79 ± 7.49%HR_max_
Netball-HIIT session	
*Netball-HIIT 1*	77.97 ± 6.55%HR_max_
*Netball-HIIT 2*	75.18 ± 8.48%HR_max_
*Netball-HIIT 3*	82.58 ± 4.79%HR_max_
*Netball-HIIT 4*	83 ± 4.62%HR_max_
*Netball-HIIT 5*	85 ± 3.77%HR_max_

**Table 3:** Participant Netball-HIIT vs. Netball Knee Program Study (heart rate, distance, affect data) (Australia, 2021).

Measure and Week No.	N	Netball-HIIT (n = 15)		Knee Program (n = 15)		One-Way ANOVA (F)	*p* Value
	Netball-HIIT: Knee Program	Mean	SD	Mean	SD		
Heart Rate 1	13:13	141.69	12.96	115.85	11.22	29.551	<0.001
Heart Rate 2	13:13	153.69	17.98	151.00	12.28	0.40	0.535
Heart Rate 3	13:13	137.15	14.94	130.46	13.70	1.42	0.245
Heart Rate 4	12:11	128.92	11.00	116.82	11.33	6.75	0.017
Heart Rate 5	11:12	134.73	11.93	121.83	10.79	7.41	0.013
Peak Heart Rate 1	13:13	169.77	16.24	143.77	11.20	22.58	0.000
Peak Heart Rate 2	13:13	153.69	17.98	151.00	12.28	0.20	0.660
Peak Heart Rate 3	13:13	156.85	16.60	162.38	17.56	0.68	0.417
Peak Heart Rate 4	12:11	138.63	46.05	148.73	13.58	0.15	0.701
Peak Heart Rate 5	11:12	159.82	16.85	153.83	12.61	0.94	0.343
Distance 1	13:13	0.40	0.13	0.54	0.30	2.12	0.158
Distance 2	13:13	0.49	0.23	0.52	0.20	0.11	0.748
Distance 3	13:13	0.80	0.26	0.80	0.28	0.00	0.981
Distance 4	12:11	0.69	0.28	0.81	0.21	0.93	0.346
Distance 5	11:12	0.73	0.24	0.84	0.02	2.44	0.133
Change Affect 1	13:14	−0.23	1.48	−0.29	1.64	0.01	0.928
Change Affect 2	15:13	−0.20	1.86	−0.15	0.90	0.01	0.936
Change Affect 3	15:13	0.27	1.28	0.46	1.45	0.14	0.709
Change Affect 4	11:11	−0.55	1.29	0.18	1.08	2.05	0.168
Change Affect 5	13:11	−0.31	1.18	−0.10	2.18	0.09	0.772

Note: Heart rate and peak heart rate expressed as beats per minute (BPM), and distance is expressed in kilometers (km). N indicates the number of participants with watch data or completed feeling state questionnaire. Note: The affective/feeling state questionnaire is a one-item questionnaire administered pre- and post-HIIT sessions. Participants respond to the question: ‘How are you feeling right now?’ (−5 = very bad to +5 = very good).

**Table 4:** Participant Netball-HIIT vs. Netball Knee Program Study (Physiological and Psychological Outcomes) (Australia, 2021).

Measure	Netball-HIIT (n = 15)			Netball Knee Program (n = 15)							
	Baseline	SD	8 Weeks Posttest	SD	Baseline	SD	8 Weeks Posttest	SD	Adjusted Difference in Change (95% CI) ^a^	*p* Value	Cohen’s *d* Effect Size
*Physiological*											
Pushup (No.)	8.07	4.85	12.08 ^b^	6.53	11.00	6.58	14.12 ^b^	7.53	1.2 (−1.13–4.37)	0.238	0.49
Jump (cm)	154.27	15.56	167.73 ^b^	18.14	153.53	22.71	163.10 ^b^	22.43	0.8 (−5.78–13.56)	0.417	0.36
20 m SRT (laps)	34.13	13.60	45.07	16.41	47.87	11.80	47.58	14.42	2.4 (1.54–20.91)	0.025	0.89

No. = number; CI = confidence interval; SD = standard deviation ^a^ Adjusted mean = difference and 95% CI between Netball-HIIT and Netball Knee Program after 6 weeks (Intervention–comparison) adjusted for baseline scores; ^b^ Significant within-group change over time *p* > 0.05; Cohen’s *d* calculated as *d* = mean difference in change scores/pooled standard deviation and interpreted as 0.2 = small effect, 0.5 = medium effect, 0.8 = large effect.

## Data Availability

Data are unavailable due to privacy or ethical restrictions.
